# Emulsion PCR: A High Efficient Way of PCR Amplification of Random DNA Libraries in Aptamer Selection

**DOI:** 10.1371/journal.pone.0024910

**Published:** 2011-09-15

**Authors:** Keke Shao, Weifeng Ding, Feng Wang, Haiquan Li, Da Ma, Huimin Wang

**Affiliations:** 1 Clinical Laboratory, Yancheng City NO.1 People's Hospital, Yancheng, China; 2 Laboratory Center, Affiliated Hospital of Nantong University, Nantong, China; Institut Jacques Monod, France

## Abstract

Aptamers are short RNA or DNA oligonucleotides which can bind with different targets. Typically, they are selected from a large number of random DNA sequence libraries. The main strategy to obtain aptamers is systematic evolution of ligands by exponential enrichment (SELEX). Low efficiency is one of the limitations for conventional PCR amplification of random DNA sequence library in aptamer selection because of relative low products and high by-products formation efficiency. Here, we developed emulsion PCR for aptamer selection. With this method, the by-products formation decreased tremendously to an undetectable level, while the products formation increased significantly. Our results indicated that by-products in conventional PCR amplification were from primer-product and product-product hybridization. In emulsion PCR, we can completely avoid the product-product hybridization and avoid the most of primer-product hybridization if the conditions were optimized. In addition, it also showed that the molecule ratio of template to compartment was crucial to by-product formation efficiency in emulsion PCR amplification. Furthermore, the concentration of the Taq DNA polymerase in the emulsion PCR mixture had a significant impact on product formation efficiency. So, the results of our study indicated that emulsion PCR could improve the efficiency of SELEX.

## Introduction

Aptamers are single-stranded DNA or RNA oligonucleotides capable of binding to other target molecules with high specificity, affinity and stability [1]–[3]. In addition, targets characteristics will be affected after binding. For instance, aptamers often bind to the functional parts of proteins and inhibit their activity [4]. These oligonucleotides fragments share most properties with monoclonal antibodies and have been showed that they successfully replaced antibodies in ELISA and Western blot [5], even smaller, more stable, and easier to be chemically synthesized compared with antibodies [6]–[7]. In particular, aptamers are very attractive as a replacement for antibodies in diagnostics and treatment of diseases. Aptamers can be obtained in vitro by directed selection from libraries of random DNA sequences. The main strategy for obtaining aptamers is designated as SELEX (systematic evolution of ligands by exponential enrichment) which consists of several rounds of sequence selection that bind to specific target molecules. For each round, it includes two stages: (1) separation of oligonucleotide-target complexes with free targets and non-bound oligonucleotides; and (2) amplification of the bound sequences by polymerase chain reaction (PCR).

Many studies showed that flow cytometry, capillary electrophoresis and other techniques are efficient methods for separation in apatamer selection [8]–[9]. However, only few reported PCR amplification application in this area. Actually, efficient separation and amplification are both necessary for productive SELEX process. However, during the PCR amplification stage, by-products formation inhibits the product generation and consequently limits the application of conventional PCR in apatamer selection. Musheev's study [10] showed that by-products appeared as early as the fifth cycle of PCR. Furthermore, when the products reached maximum level, additional five cycles completely converted the products to by-product even though the primers were still present with certain concentration in the mixture, which leaded to the loss of potential high affinity and specificity apatamers and finally, even the failure of selection. They thought the most likely mechanism of this conversion is product–product hybridization rather than primer-primer hybridization.

**Figure 1:**
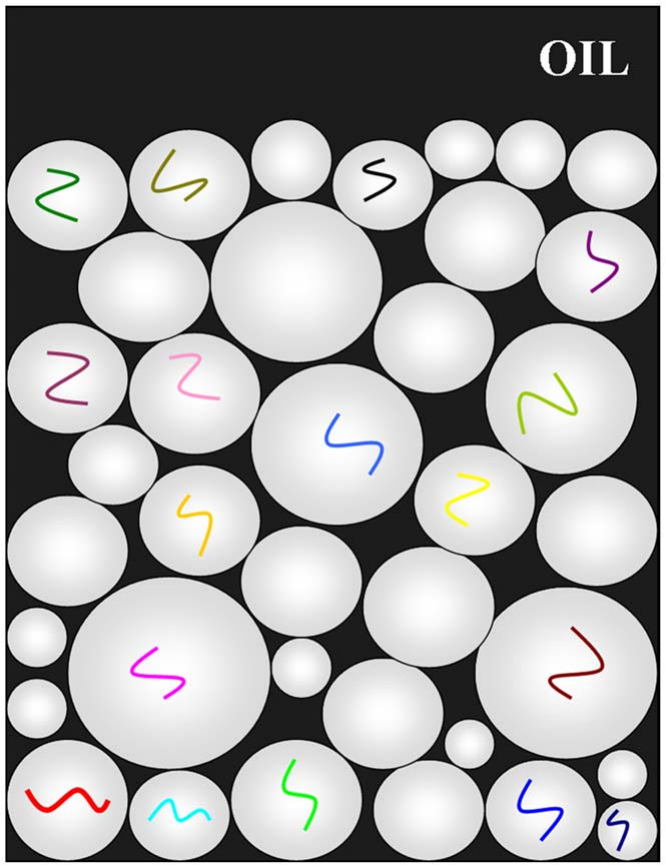
Simulation diagrams of emulsion PCR amplification of a random DNA library. Different compartment contained different templates marked with different colors, and some compartment are empty.

Therefore, we proposed that product–product hybridization could be avoided if partition the reaction mixture into droplets, each of which is designed to contain only one template. Emulsion PCR complex contains millions of cell-like compartments which are separated from each other without exchange of macromolecules, especially the PCR products (Fig. 1). Emulsion PCR is a single-molecule PCR [11]. Emulsion PCR amplification of complex gene libraries could prevent the absence of chinmeric products from happening [12]. Tatjana Schütze demonstrated emulsion PCR could be used in SELEX experiments for an unbiased amplification [13]. Nowadays the most diverse libraries are used in SELEX experiments where highly variable initial nucleic acid pools of up to 10^15^ individual sequences are employed [1], [2]. How to make the emulsion PCR amplification of a random DNA library to achieve the optimum results? How to make the emulsion PCR completely avoid the formation of by-products? Thus the characteristic of emulsion PCR and the mechanisms of by-products forming are necessary to study.

Traditionally, Polyacrylamide and Agarose Gel Electrophoresis are used for qualitative PCR products analysis while the Capillary Electrophoresis and Microfluidic Chip Electrophoresis, more accurate and reproducible methods, are used for quantitative test [14]–[15]. We used polyacrylamide gel electrophoresis to analyze PCR products, which is more sensitive and accurate than agarose gel electrophoresis, and microfluidic chip electrophoresis to quantitate PCR products, which is easier than capillary electrophoresis.

The aims of this study were to: 1.examine whether emulsion PCR can reduce by-product formation and increase the products formation; 2. explore the characteristics of emulsion PCR used in aptamer selection. 3. study the mechanism of by-products forming.

## Materials and Methods

### Chemicals and materials

Chemicals and materials included DreamTaq DNA polymerase (Fermentas, Lithuania); dNTP mixture solution (Sangon Biotech, Shanghai, China); DNA template (Integrated DNA Technologies, Coralville, IA); primers (Sangon Biotech, Shanghai, China); Span 80, Tween 80, Triton X-100, mineral oil and ether (Sigma). All solutions were prepared by using Milli-Q-quality deionized water and filtered through a 0.22 µm filter (Millipore, Nepean, Canada).

### DNA primers and library

The library was constructed as a single-stranded 90-mer oligonucleotides with the following sequence: 5′-GAACATTGGCGTCCGTGAG-N52-ACTTCCTCAAACGCCCAA-3′, where the central 52 bp contained random sequence (N) based on equal incorporation of A, G, C, and T at each position. A set of primers was used: up-primer, 5′-GAACATTGGCGTCCGTGAG-3′ and lo-primer, 5′-TTGGGCGTTTGAGGAAGTG-3′.

### Emulsions of PCR

Water-in-oil emulsions for PCR were prepared according to the previously described method with minor modifications [16]. The oil phase, freshly prepared each day, was composed of 4.5% Span 80, 0.4% Tween 80, and 0.05% Triton X-100 in mineral oil. The aqueous phase was a PCR mixture. Water-in-oil emulsions were prepared by adding the ice-cooled PCR reaction mixture (0.1 ml) gradually (in 10 aliquots of 10 µl over 2 min) to the oil phase (0.2 ml) in a 2 ml round-bottom cryogenic vial whilst the mixture was continuously stirred at 1,500 rpm with a magnetic microstir bar (no. 58948-377, VWR Scientific) on a VWR model 625 magnetic for 5 min before PCR cycling.

### PCR

Conventional PCR mixture was prepared as described below: 0.4 µmol/L of each primer, 2.5 mmol/L MgCl_2_, 0.2 mmol/L dATP, 0.2 mmol/L dCTP, 0.2 mmol/L dGTP, 0.2 mmol/L dTTP, 0.05 unit/L Taq DNA polymerase, and 0.01 pmol/ml ssDNA template were added into the DreamTaq Buffer to adjust total volume to 50 µl. The emulsion PCR mixture was prepared as described below: 0.4 µmol/L of each primer, 3.5 mmol/L MgCl_2_, 0.4 mmol/L dATP, 0.4 mmol/L dCTP, 0.4 mmol/L dGTP, 0.4 mmol/L dTTP, 0.125 unit/L Taq DNA polymerase, and 0.01 pmol/ml ssDNA template were added into the DreamTaq Buffer to adjust total volume to 100 µl. PCR amplification was performed on PTC-200 Peltier Thermal Cycler (MJ Research, USA). All PCR procedures were carried out under the following cycling conditions: 94°C for 2 min, some cycles of 94°C for 30 sec, 65°C for 30 sec, and 72°C for 30 sec. In the optimizing experiments, the concentration of template ranged from 0.002 to 2 pmol/ml, the annealing temperature ranged from 50°C to 70°C, the concentration of Taq DNA polymerase ranged from 0.025 to 0.175 units/L, the concentration of primers ranged from 0.4 to 1.2 mmol/L, and the cycles ranged from 10 to 40.

### Recovery of the reaction mixture

The water-in-oil emulsion from the PCR tube was pooled and spun at 9000 g for 5 min to remove the oil phase, leaving the concentrated (but still intact) emulsion at the bottom of the vial. Two volumes of water-saturated ether were added to one volume of the concentrated emulsion, and the mixture was vortexed and centrifuged briefly to remove the ether phase. The aqueous phase was washed two times with ether and dried at room temperature.

### DNA assays

Polyacrylamide gel electrophoresis silver staining and Microfluidic chip electrophoresis were used for analysis of the PCR products. DNA1000 Kit (Agilent Technologies, Germany) was used and all chips were prepared according to the manufacturer's instructions. The Agilent clipper 1000 Bioanalyzer software automatically displayed electropherograms and calculated the size and concentration of each separated band.

## Results

### Emulsion PCR amplification of a random DNA library versus conventional PCR amplification of a random DNA library

It is known that, in conventional PCR amplification of a homogeneous DNA sample, quantity of product increases exponentially to the maximal level then stay at the plateau phase. However, for PCR amplification of Random DNA libraries, it reached the maximal level first and then dropped rapidly to 0 [10]. In this study, we compared the formation of products and by-products in conventional PCR with them of emulsion PCR amplification of a random DNA library. Conventional PCR was carried out for 10, 12, 14, 16, 18, 20, 22, 24, 26, 28, 30 and 32 cycles separately and Emulsion PCR was carried out for 10, 15, 20, 25, 30, 35 and 40 cycles individually.

**Figure 2:**
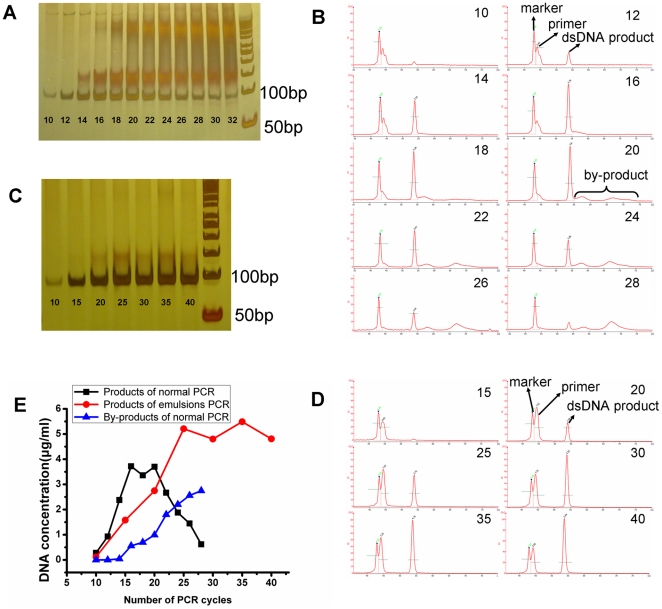
Conventional PCR amplification of a DNA random library versus emulsion PCR. PAGE electropherograms of PCR reaction mixtures for amplification of a random DNA library with conventional PCR (A) and emulsion PCR (C). Microfluidic chip electrophoresis electropherograms of PCR reaction mixtures for amplification of a random DNA library with conventional PCR (B) and emulsion PCR (D). Dynamics of product and by-product concentrations in conventional PCR and emusion PCR (E). Conventional PCR amplification was performed with cycles ranging from 10 to 32; the template concentration was 0.01 pmol/ml; the primer concentration was 0.4 µmol/L; and the concentration of Taq DNA polymerase was 0.05 U/µl. Emulsion PCR amplification was performed with cycles ranging from 10 to 40; the template concentration was 0.01 pmol/ml; the primer concentration was 0.4 µmol/L; and the concentration of Taq DNA polymerase was 0.125 U/µl.

Through PAGE electropherograms of the PCR reaction mixture, we found the product formation increased at the beginning and then decreased rapidly, and the by-product formation began to appear at 14 cycles and increased along with the progression of the PCR cycle in conventional PCR (Fig. 2A). On the contrary, in emulsion PCR, we found the product formation reaches the maximal level and then stayed at the plateau phase. The by-product formation was found at 25 cycles, the amount of which was relative small and did not increase with the addition of PCR cycles (Fig. 2C).

Through microfluidic chip electrophoresis electropherograms of the PCR reaction mixture, we found the same condition in conventional PCR (Fig. 2B). But for emulsion PCR, by-product can't be detected (higher than 0.02 µg/ml was recognized as a valid peak) (Fig. 2D).

Fig. 2E shows the dynamic concentration of the products and by-products in conventional PCR and emulsion PCR. When the conventional PCR amplification increased to 28 cycles, products formation curve showed the form of parabola. The peak concentration (3.72 µg/ml) appeared at 16 cycles. However, the concentration of the products was 0.62 µg/ml at 28 cycles. In view of the by-products formation increasing constantly when they appeared at the 14th cycle, the identification of appropriate PCR cycles is necessary if the maximal copies of products are needed in conventional PCR. On the contrary, products formation by emulsion PCR reached the most (5.21 µg/ml) at 25 cycles which was much higher than the peak concentration of conventional PCR, followed by a platform period. It showed a similar amplification pattern as conventional PCR amplification of a homogeneous DNA sample.

### Effects of template concentrations

The influence of concentrations of the template on the yield of the products and by-products in emulsion PCR amplification of a random DNA library was measured. In 50 µl aqueous phase, the concentration of template was 0.002 pmol/ml, 0.01 pmol/ml, 0.02 pmol/ml, 0.2 pmol/ml and 2 pmol/ml separately. Emulsion PCR was carried out for 35 cycles, by which could enable PCR to reach the platform phase.

**Figure 3:**
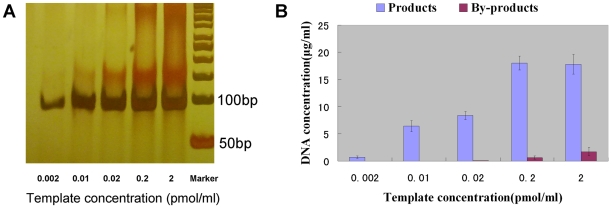
Influence of the number of template molecules. Panel A indicates the PAGE electropherograms of PCR reaction mixtures for amplification of a random DNA library with different amounts of template molecules. Panel B indicates the concentration of dsDNA products and by-products obtained from microfluidic chip electrophoresis. Emulsion PCR amplification was carried out for 35 cycles; the annealing temperature was 65°C; the primer concentration was 0.4 µmol/L; and the concentration of Taq DNA polymerase was 0.125 U/µl.

The copy number of products increased with the concentration of the template increasing within the range between 0.002 pmol/ml and 0.2 pmol/ml. With higher amount of template (2 pmol/ml), the output of products was less than that of lower template at 0.2 pmol/ml (Fig. 3B), suggesting that some products converted to by-products. For 0.02 pmol/ml template, only a small amount of by-products can be detected. After this point, the by-products increased with more template molecules (Fig. 3A, Fig. 3B). The mean droplet diameter of Emulsion particles was 4 µm as measured by light microscopy and laser diffraction spectroscopy. It showed that 100 µl aqueous phase could form about 3×10^9^ compartments and the ratio of various copy number of templates (0.002 pmol/ml, 0.01 pmol/ml, 0.02 pmol/ml, 0.2 pmol/ml and 2 pmol/ml) to number of compartments is 1∶25, 1∶5, 2∶5, 4∶1and 40∶1. Therefore, we concluded that the more different templates in one compartment, the more by-products would form. For 2 pmol/ml template, theoretically, one compartment could contain 40 different templates on average. So, there is high possibility for them to form by-products. Therefore, 0.01 pmol/ml template in 100 µl aqueous phase, equals to 1∶5, was used as the best molar ratio of number templates to compartments, to guarantee reach comparative high amount of product and low level of by-products.

### Effects of annealing temperature

**Figure 4:**
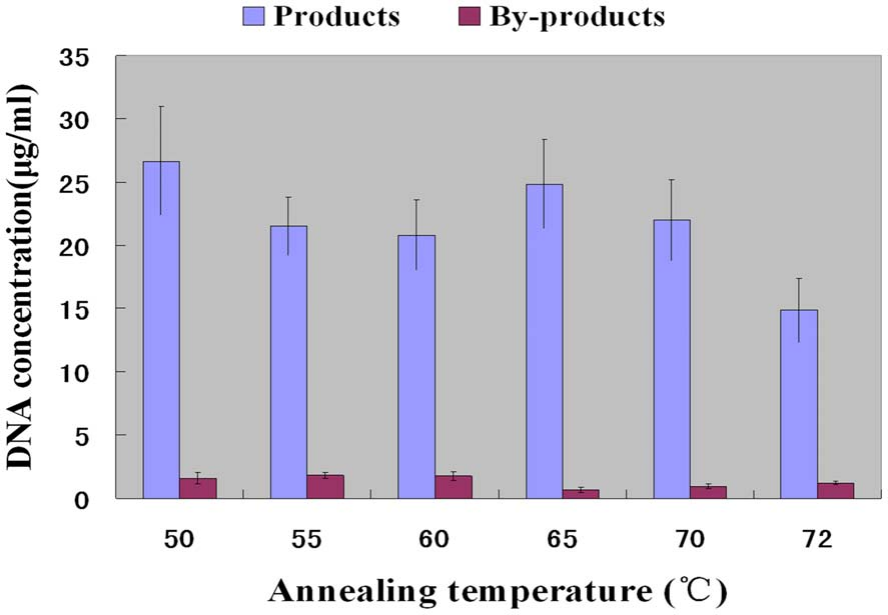
Influence of the annealing temperature. The concentration of the dsDNA products and by-products was obtained from microfluidic chip electrophoresis. Emulsion PCR amplification was carried out for 35 cycles; the number of template was 0.01 pmol/ml; the primer concentration was 0.4 µmol/L; and the concentration of Taq DNA polymerase was 0.125 U/µl.

Normally, in certain range, increasing annealing temperature can improve the specific binding of primer and template and also reduce the by-products formation. In this study 2 pmol/ml template, normally generating a large amount of by-products (Fig. 3B), was used to study the influence of annealing temperature on the yield of the products and by-products in emulsion PCR amplification of a random DNA library. Emulsion PCR was carried out for 35 cycles with annealing temperature from 50 to 72°C. From 50 to 70°C, product output didn't show significantly change. In addition, the amount of by-products decreasing with the annealing temperature increasing was not observed (Fig. 4). However, products yield significant decreased at 72°C annealing temperature (Fig. 4). These results indicated that annealing temperature has no significant influence on by-product formation in emulsion PCR.

### Effects of DNA polymerase concentrations

**Figure 5:**
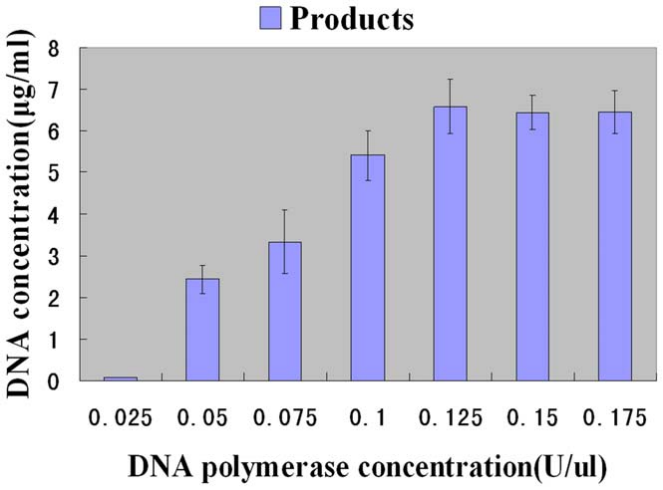
Influence of DNA polymerase concentrations. Emulsion PCR amplification was carried out for 35 cycles; the number of templates was 0.01 pmol/ml; the primer concentration was 0.4 µmol/L; and the annealing temperature was 65°C.

In view of the best molar ratio of templates to compartments is 1∶5, 80% compartments did not contain a template DNA to form a complete PCR system and therefore had no product formation. Theoretically, about 80% materials were wasted in the PCR process and the product may decrease correspondingly. So, what if each compartment has enough materials for PCR? Whether the products output would increase tremendously? In this study, the effect of DNA polymerase and primers concentrations on product and by-product outputs was tested. The effect of DNA polymerase was first studied. Emulsion PCR was carried out for 35 cycles with 0.4 mmol/L dNTP, 0.4 µmol/L of each primer and 0.01 pmol/ml templates, while the concentration of DNA polymerase varied between 0.025 and 0.15 U/µl. The annealing temperature was 65°C. It was found that the yield of the products increased gradually from 0.07 µg/ml to 6.58 µg/ml with the higher concentration of DNA polymerase. It reached the maximal level at 0.125 U/µl and followed by a platform period (Fig. 5). Meanwhile, the by-products could not be detected by Microfluidic chip electrophoresis. Therefore, 0.125 U/µl DNA polymerase was chosen as the best concentration for emulsion PCR.

### Effects of primer concentrations

**Figure 6:**
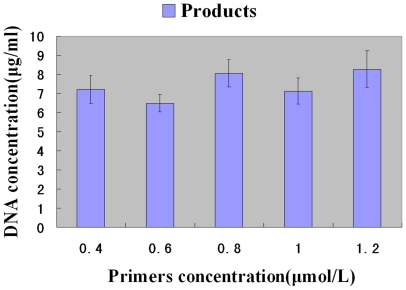
Influence of primer concentrations. Emulsion PCR amplification was carried out for 35 cycles; the number of templates was 0.01 pmol/ml; the concentration of Taq DNA polymerase was 0.125 U/µl; and the annealing temperature was 65°C.

Then the influence of primer concentrations on the yield of products and by-products was studied. Emulsion PCR was carried out for 35 cycles with 0.4 mmol/L dNTP, 0.125 U/µl DNA polymerase and 0.01 pmol/ml templates, while the concentration of primers varied between 0.4 µmol/L and 1.2 µmol/L. The annealing temperature was 65°C. It was found that the amount of product did not increase with the concentration of primer increasing. However the yield of products were all higher than 6.5 µg/ml, while by-product cannot be detected by Microfluidic chip electrophoresis (Fig. 6). Thus we chose 0.4 µmol/L as the best concentration for this emulsion PCR, because it could best save primers and get enough products.

### The mechanisms of by-products forming in conventional PCR

**Figure 7:**
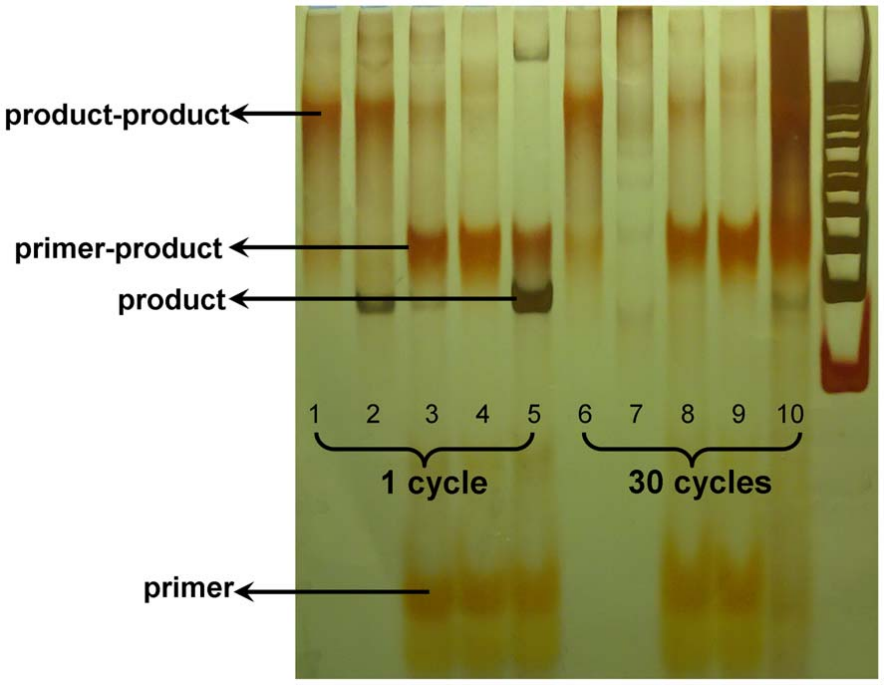
By-product forming. Conventional PCR was carried out for 1 and 30 cycles with 5 µg/ml template. 1 and 6: PCR mixture only include template. 2 and 7: PCR mixture include all except primer. 3 and 8: PCR mixture include all except dNTP. 4 and 9: PCR mixture include all except DNA polymerase. 5 and 10: PCR mixture include all the masteries.

Finally, by-product formation mechanism was studied. Conventional PCR was carried out for 1 and 30 cycles with 5 µg/ml template that were enough for analysis if no template was amplified. It was found that when PCR mixture only includes template, after 1 cycle, the templates were completely converted to by-products which were only formed by product-product hybridization, the same situation appeared after 30 cycles (Fig. 7). When PCR mixture includes all except primer, after 1 cycle, most of templates were converted to by-products which were only formed by product-product hybridization too, and all templates were converted to by-products after 30 cycles. When PCR mixture includes all except dNTP, after 1 cycle, most of templates were converted to by-products, most of which were formed by primer-product hybridization (Fig. 7), and all templates were converted to by-products after 30 cycles (Fig. 7). The same situation appeared when PCR mixture includes all except DNA polymerase (Fig. 7).

## Discussion

By-product formation is a big obstacle for effective conventional PCR amplification of a random DNA library in aptamer selection. In this study, emulsion PCR was used to resolve this problem. We observed a smear going up from the product band on the PAGE and microfluidic chip electrophoresis of conventional PCR (Fig. 2A, 2B, 2C, 3A), indicating that all the by-products were longer than dsDNA products. Musheev et al [10] hypothesized that by-products were formed from product-product hybridization. In the mixture of emulsion PCR, there are millions of cell-like isolated compartments as independent amplification system without affect each other. And the product–product hybridization may be maximally avoided. The result indicated that, by-product output significant decreased, and could not be detected by microfluidic chip electrophoresis. However, it showed that the more different templates in one compartment, the more by-products would form. When the amount of the template was 10^−1^ pmol, one compartment could contain 40 different templates. Therefore there is high possibility to form by-products (Fig. 3A, 3B). And the conventional PCR was equivalent to one compartment containing about 10^9^ different templates, thus the by-product in conventional PCR was inevitable. When there is no primer in PCR mixtures, the by-products were formed by product-product hybridization. When there is no dNTP or DNA polymerase in PCR mixtures, most of the by-products were formed by primer-product hybridization which the electrophoretic speed was faster than product-product (Fig. 7). Thus in conventional PCR amplification of a random DNA library, the by-products first formed by primer-product hybridization. When the cycles increased, the concentration of primer decreased and product-product hybridization began to increase (Fig. 2A). So emulsion PCR can prevent the product-product hybridization because different kinds of products were separated in different compartments. But it can not stop all of the primer-product hybridization, even though there is only one template in each compartment (Fig. 2C).

In SELEX, if the PCR products with high rate of hybridization was the good candidates for aptamer with affinity and high specificity, the selection result wouldn't be optimistic. Emulsion PCR produces higher output and type of products and lower level of by-products than conventional PCR and lead to more effective SELEX. In addition, it could also simplify PCR amplification of a random DNA because of the avoidance of over-amplification (Fig. 2C) which even leads to a complete loss of the products in conventional PCR (Fig. 2A).

Furthermore, for the first time, we studied the features of emulsion PCR amplification of random DNA libraries in aptamer selection. This study showed the best way to control the by-product was to choose the best ratio of number of templates to number of compartments. 1∶5 was chosen as the best ratio, which could avoid product-product hybridization completely (Fig. 3A). And the study also indicated that annealing temperature has no significant influence on by-product formation in emulsion PCR. Thus the ratio of number of templates to number of compartments was the key factor on by-product formation in emulsion PCR.

For the amplification of a random DNA library, higher concentration of DNA polymerase were used for emulsion PCR compared with conventional PCR which is usually lower than 0.1 U/µl [10]. And the conventional PCR amplification of homogeneous DNA samples would decrease of the products and increase of the by-products if the concentration of DNA polymerase exceeded 0.025 U/µl [17]. When the concentration of DNA polymerase was varied from 0.025 U/µl to 0.125 U/µl, the yield of the products increased gradually from 0.07 µg/ml to 6.58 µg/ml (Fig. 5). But when the concentration of primer was varied from 0.4 to 1.2 µmol/L, the yield of the products was all higher than 6.5 µg/ml (Fig. 6), which was enough for apamer selection. Thus the concentration of primer had no significant influence on apamer selection. The same situation was happened on the concentration of dNTP(data not supply). Thus this study showed that the concentration of DNA polymerase was the key factor on product formation in emulsion PCR. This study also showed that the microfluidic chip electrophoresis was a powerful tool to optimizing PCR of random DNA libraries.

This work suggests that emulsion PCR could improve the efficiency of SELEX through improving the efficiency of PCR amplification of random DNA libraries.
